# The role of Gestational Diabetes Mellitus and pelvic floor 3D-ultrasound assessment during pregnancy predicting urinary incontinence: a prospective cohort study

**DOI:** 10.1186/s12884-023-05932-8

**Published:** 2023-09-05

**Authors:** Carlos Izaias Sartorao Filho, Sthefanie K. Nunes, Adriely B.M. Magyori, Iracema M.P. Calderon, Angelica M.P. Barbosa, Marilza V.C. Rudge

**Affiliations:** 1 Department of Gynecology and Obstetrics, Botucatu Medical School, São Paulo State University (UNESP), Benedito Spinardi st 1440, Botucatu, Assis- São Paulo State 19815-110 Brazil; 2 Educational Foundation of Assis Municipality (FEMA), Medical School, Assis, Brazil; 3 School of Philosophy and Sciences, Department of Physiotherapy and Occupational Therapy, São Paulo State University, Marília, Sao Paulo Brazil

**Keywords:** Pelvic floor, Gestational diabetes Mellitus, Urinary incontinence, Ultrasound

## Abstract

Postpartum urinary incontinence may have a severe impact on women’s health. Despite pregnancy and parturition being the most recognized risk factors, methods to identify new pregnant predictor risk factors are needed. Our study investigated the Gestational Diabetes Mellitus, clinical and pelvic floor 3D-ultrasound markers in pregnant women as predictors for 6–18 months of urinary incontinence. This prospective cohort study included nulliparous pregnant women submitted to Gestational Diabetes Mellitus screening in the second trimester. Pelvic floor 3D Ultrasound was performed at the second and third trimesters of gestation to evaluate the pelvic floor muscles and functions. Clinical data, the ICIQ-SF, and ISI questionnaires for urinary incontinence were applied in the third trimester and 6–18 months postpartum. Univariate analysis (P < .20) to extract risk factors variables and multivariate logistic regression analysis (P < .05) to obtain the adjusted relative ratio for urinary incontinence were performed. A total of 93 participants concluded the follow-up. Using the variables obtained by univariate analysis and after adjustments for potential confounders, multivariate analysis revealed that Gestational Diabetes Mellitus exposure was a solid and independent risk factor for 6–18 months of urinary incontinence (Adjusted RR 8.08; 95%CI 1.17–55.87; P:0.034). In addition, a higher Hiatal area observed in distension maneuver from the second to third trimester was negatively associated (Adjusted RR 0.96; 95%CI 0.93–0.99; P:0.023). In conclusion, Gestational Diabetes Mellitus was positively associated with 6–18 months of urinary incontinence, and higher Hiatal area distension was negatively associated.

## Introduction

Gestational diabetes mellitus (GDM) significantly increases the adverse maternal and perinatal outcomes and the likelihood of type 2 Diabetes Mellitus, cardiovascular disease, and Urinary Incontinence (UI) [1–3]. GDM and UI combination seems to have hyperglycemic myopathy of the Pelvic Floor Muscle (PFM) and Rectus Abdominis Muscle (RAM) as the underlying mechanism [1, 4–9]. Current clinical and experimental studies suggest that Pregnancy Specific-UI (PS-UI) is the earliest symptom of hyperglycemic PFM and RAM myopathy during pregnancy, despite overlooked complaints during prenatal care assistance [5, 6, 8–16]. Nevertheless, a previous study showed that the PS-UI, the severity of long-term UI, and the negative impact of UI on quality of life are more significant among GDM pregnant women in the first year postpartum [9]. However, none of these studies correlated the clinical pregnancy data and the anatomical and functional PFM ultrasound assessment during the pregnancy as predictors of postpartum UI [9]. The translabial three-dimensional ultrasonography (3DUS) provides an adequate and reliable evaluation of anatomical and functional PFM status [17]. Besides, we previously demonstrated that GDM-pregnancy had lower Hiatal Area (HA) biometry changes from 24 to 38 weeks of gestation [7]. Therefore, it seems highly likely that GDM alters the maternal adaptation during the pregnancy of PFM structures, leading to a decreased distensibility of the PFM at the end of the third trimester [7]. Reports confirm that GDM and UI are two clinical entities with substantial social and economic burdens associated with significant direct and indirect public health costs [18, 19]. Despite these observations, the link between GDM and long-term UI remains mostly unexplored. Thus, developing 3DUS markers of hyperglycemic myopathy during pregnancy as predictors of long-term UI is necessary to further intervention propositions. Moreover, developing a predictive UI test during pregnancy collaborates to identify a high-risk population accurately. We hypothesized that GDM damages the PFM during pregnancy [7, 20, 21]. This study aimed to identify clinical and PFM 3DUS markers in the second and third trimesters of GDM and non-GDM pregnancy as 6–18 months UI predictors.

## Methods

This prospective non-interventional cohort study within the prospective Diamater cohort study protocol [4] started in 2015 at the Perinatal Diabetes Research Center (PDRC), Botucatu Medical School-UNESP. Our institution’s GDM rate is 30–40% due to a tertiary referral hospital.

Regulatory approval was obtained from the Institutional Review Board of Botucatu Medical School (#1.716.895). Written informed consent was obtained from all participants, and all issues related to medical ethics met the requirements of the Helsinki Declaration. All patients involved were properly handled for their morbidities.

Nulliparous singletons over 18 years of age, submitted to GDM screening and translabial 3DUS at two-time points in the second and third trimesters of pregnancy, were enrolled. All women included in this study received prenatal care at PDRC. Exclusion criteria were loss to follow-up, pre-gestational diabetes, pre-gestational UI, severe comorbidities, inadequate GDM screening, preterm birth, use of hypoglycemic drugs in the pregnancy, or new pregnancy during the postpartum period. We also considered only pregnant women with mild GDM, regularly controlled with nutritional and lifestyle interventions, submitted to planned C-sections - the majority based on-demand particular decision, and performed before the labor onset.

Eligible women were included between 24 and 28 weeks of gestation. At enrollment, women completed a detailed questionnaire on sociodemographic information, past medical history, regular physical activity (150 min/week), tobacco use, initial pregnancy weight in Kg, height in cm, and Body Mass Index (weight in kilograms divided by height in meters squared). Ethnicity was defined as Caucasian or non-Caucasian. The researcher performed a PFM 3DUS at 24–28 and 36–40 weeks of gestation (CISF). The translabial 3DUS raw data were stored for posterior blinded and offline analysis. The follow-up continued until April 2020. The same clinical and anthropometric evaluation was done at 36–40 weeks.

Data regarding fasting plasma glucose in the first trimester, 75 g-Oral Glucose Tolerance Test (OGTT) in the second trimester of pregnancy, gestational age (in weeks) at the two-time points of 3DUS, the ISI, and the ICIQ-SF questionnaire scores for PS-UI at 36–40 weeks of pregnancy were recorded.

At 6–18 months after a planned C-section, breastfeeding duration, regular physical activity (minimum 150 min/week), postpartum smoking, newborn weight in grams, and gestational age at delivery, to classify newborn as appropriate, large or small for gestational age, and urinary incontinence (yes or not) with ISI and ICIQ-SF scores were collected.

The primary outcome was the prevalence of 6–18 months of UI development after a planned C-section. We considered the C-section a protective event, avoiding the supposed adverse effects of the parturition and vaginal birth on the pelvic floor. For this purpose, we excluded women exposed to the active labor onset and vaginal birth. We investigated the association between the primary outcome measure and the risk factors, including maternal and perinatal characteristics, the first-trimester fasting glucose negative and the OGTT results as positive or negative for GDM diagnosis, the Pregnancy Specific UI occurrence, and severity of PS-UI, and the PFM 3DUS structural and functional biometry at two-time points during pregnancy.

Women in the study met the Fasting Blood Glucose (FBG) criteria in the first trimester (1– 13 gestational weeks) lower than 92 mg/dL or 5.1 mmol/L [22]. All pregnant women underwent 75-g OGTT between 24 and 28 weeks. The cutoffs were 92, 180, and 153 mg/dL (5.1, 10.0, and 8.5 mmol/L) at fasting, one and two hours after glucose overloading. The GDM was defined by one or more values equal to or more than the respective cutoffs [23–25]. A glucose profile was performed during a 1-day hospital stay with the woman on a 30 kcal/Kg-diet fractionated in five meals to diagnose mild gestational hyperglycemia. Plasma glucose was measured every two hours, from 8 AM to 6 PM. The thresholds were 90 mg/dL (5.0 mmol/L) for fasting (8 h) and 130 mg/dL (7.22 mmol/L) for any postprandial level. To maintain normoglycemia, all pregnant women received the appropriate nutritional and lifestyle interventions as the cornerstone of treatment during the last pregnancy. After these instructions, when glycemia remains elevated after 1–2 weeks of lifestyle interventions, daily glucose testing was continued, pharmacological treatment was initiated, and the patient was excluded from this study.

PS-UI was defined as any urinary leakage that started during pregnancy [26]. One specific question was asked to all pregnant women about urinary leakage that started during this pregnancy. If yes, the patient was classified as having PS-UI. Pregnant women with PS-UI complete the UI occurrence and severity evaluation with ISI [27] and ICIQ-SF [20, 28] surveys at 36–40 weeks of gestation [29].

We performed the US examination according to the protocol proposed by Dietz et al. [17] at two-time points in pregnancy: 24–28 weeks (T1) and 36–40 weeks (T2). The functional maneuvers of contraction and distension were previously explained to the participants. The variables of 3DUS in T1 and T2, at rest, contraction, and distension, were collected offline after completing the study by the same observer, blinded (CISF). The measurements: Hiatal area in square cm; Antero-Posterior (AP) diameter in cm; Transversal diameter in cm; variation of AP from T1-T2 in cm; variation of Hiatal area T1-T2 in square cm; variation of Transversal diameter from T1-T2 in cm; the relationship between rest and contraction in T1, in T2 and the T1-T2 variation from all biometry; the relationship between rest and Valsalva in T1, T2, and the T1-T2 variation; the relationship between contraction and Valsalva in T1, T2, and the T1-T2 variation; the percentual variation from each biometry analysis were calculated [7, 30]. We used US equipment with a volumetric curved array three-dimensional transducer in the sagittal plane and 85° in the coronal plane. The exposition and the follow-up period were at the endpoint at 24–28 weeks of gestation (T1), 36–40 weeks (T2), birth, and 6–18 months postpartum.

The Sample Size Calculation used Cochran’s equation together with a population correction. Our estimated population size during this survey was 300 pregnant women. Thus, using a Precision Level of 5%, a Confidence Level of 95%, an estimated proportion of 10% of the exposed population, and a population size of 300 pregnant women, the appropriate sample size was 95.

Descriptive analysis was applied for the demographic characteristics described as mean, standard deviation (SD), and Minimum / Maximum (Min/Max) values and analyzed by an independent sample Student’s t-test. We first used the Cox univariate logistic regression analysis to test the association between each risk factor and long-term UI development. Then, factors with a *P*-value under 0.20 on the univariate analysis were included in a multivariate logistic regression analysis, using the backward stepwise method to test for independent associations between risk factors and long-term UI development. All significant maternal-pregnancy characteristics and the PFM 3DUS measurements from the univariate analysis were used as predictors. Finally, after adjustments for potential confounding factors, a multivariate logistic regression analysis was performed to identify the relationship between long-term UI and clinical plus PFM 3DUS at the second and third trimesters of pregnancy. Relative risk (RR) and 95% confidence intervals (95%CI) were reported. The RR and 95%CI were calculated for each clinical and 3DUS measure and adjusted for all variables. These variables were excluded successively (models 1 to 8) until reaching the best model, which was defined by the impossibility of excluding any other variable without significant loss in adjustment. A *P*-value under 0.05 was considered statistically significant for all multivariate logistic tests.

## Results

**Fig. 1:**
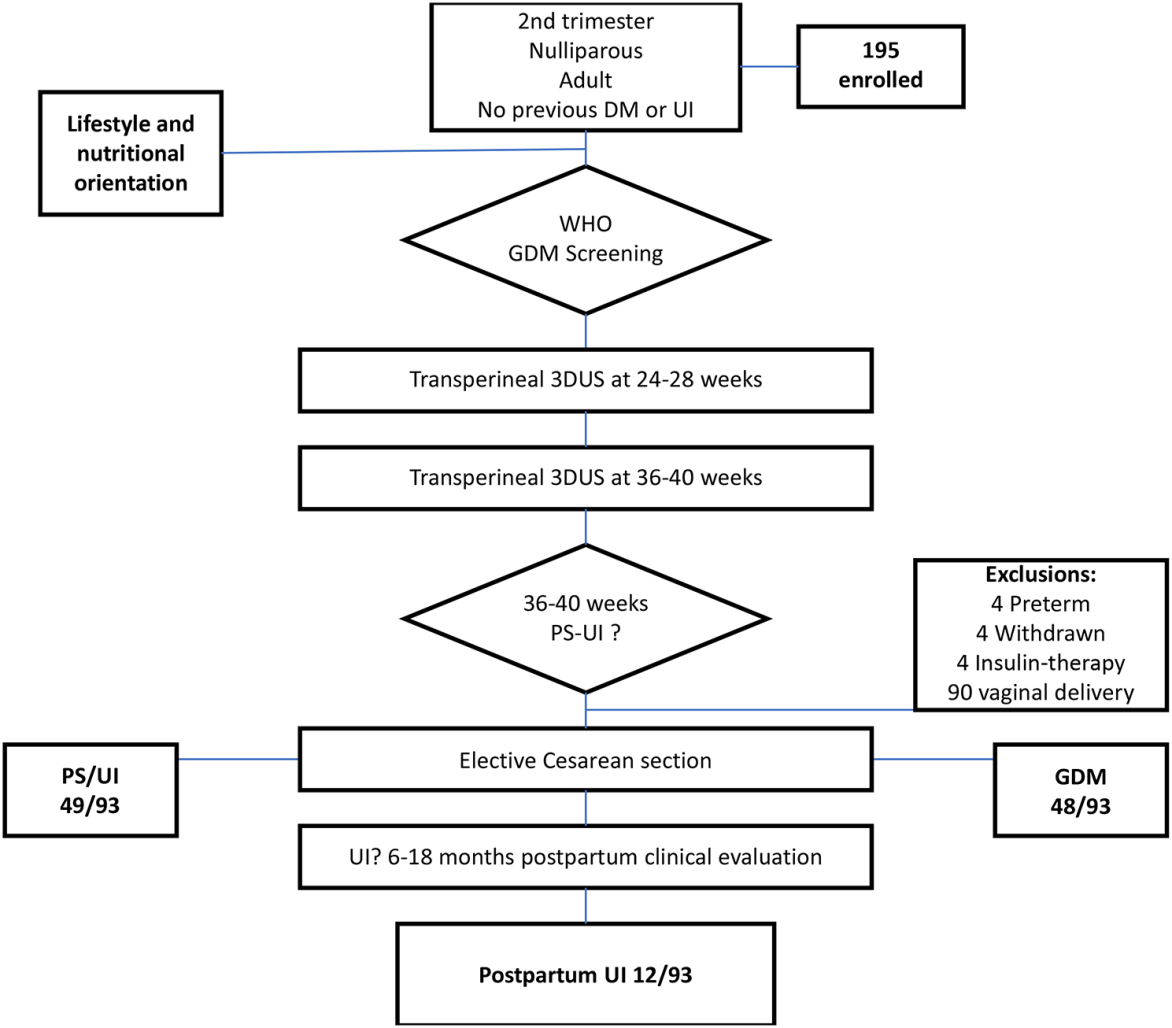
Study flow chart

One hundred ninety-five participants were enrolled. We excluded four participants due to preterm birth, four withdrawn during prenatal care, four because of insulin therapy, and ninety participants due to vaginal birth or active labor exposition. After 6–18 months postpartum, 93 participants met the eligibility criteria. Figure 1 represents the flow chart of the study, and Table 1 represents the baseline characteristics of the participants.

**Table 1:** Baseline Clinical Characteristics of the Overall Participants: N = 93

	MEAN	SD	MIN	MAX
**Age (years)**	**26.4**	**4.8**	**18.0**	**38.0**
**First-trimester maternal weight (kg)**	**70.2**	**15.8**	**44.3**	**117.6**
**First trimester Body Mass Index**	**26.2**	**5.3**	**17.6**	**38.4**
**Pregnancy weight gain (kg)**	**10.0**	**4.9**	**-0.1**	**24.8**
**Gestational age at T1 (weeks)**	**26.9**	**1.0**	**24.7**	**28.0**
**Gestational age at T2 (weeks)**	**36.4**	**0.7**	**35.6**	**38.7**
**T1-T2 interval (weeks)**	**9.6**	**1.1**	**7.6**	**13.0**
**FB Glucose - first trimester (mg/dl)**	**83.6**	**9.1**	**62.0**	**105.0**
**75 g-OGTT-fasting (mg/dl)**	**81.5**	**12.2**	**63.0**	**124.0**
**75 g-OGTT-1 h (mg/dl)**	**128.2**	**35.2**	**71.0**	**211.0**
**75 g-OGTT-2 h (mg/dl)**	**115.0**	**26.8**	**59.0**	**182.0**
**Gestational ICIQ-SF score**	**4.9**	**5.5**	**0.0**	**16.0**
**Gestational ISI score**	**2.13**	**2.28**	**0.00**	**6.00**
**Newborn weight (kg)**	**3.143**	**0.394**	**1.900**	**3.990**
**6–18 months postpartum weight (kg)**	**74.07**	**17.48**	**48.00**	**136.00**
**Breastfeeding (months)**	**6.97**	**3.49**	**0.00**	**12.00**
**6–18 months postpartum ICIQ-SF score**	**1.48**	**4.04**	**0.00**	**18.00**

SD: Standard Deviation

Min: Minimum value; Max: Maximum value

T1: 24–28 weeks of gestation; T2: 36–40 weeks of gestation; T1-T2: the period between T1 to T2

FB Glucose: Fasting Blood Glucose

ICIQ-SF: International Consultation on Incontinence Questionnaire - Short Form.

Gestational ISI: Incontinence Severity Index during pregnancy.

The mean age was 26.4 years (SD 4.8), the prepregnancy body weight was 70.2 Kg (15.8), weight gain was 10.0 Kg (4.9); the mean gestational age at T1 was 26.9 (1.0), at T2 was 36.4 (0.7), and the interval T1-T2 was 9.6 (1.1) weeks; the mean of fasting glucose at the first trimester was 83.6 (9.1) mg/dl; the 75 g-OGTT fasting (mg/dL), one hour and two hours were 81.5(12.2); 128.2 (35.2);115.0 (26.8) respectively. The ICIQ-SF gestational score was 4.9 (5.5), and ISI gestational score was 2.13 (2.28). The newborn weight was 3143 g (394), the maternal postpartum weight was 74.07 (17.49), the breastfeeding duration was 6.97 (3.49) months, and the postpartum ICIQ-SF score was 1.48 (4.04).

Table 2 reports the 3DUS measurements at rest, contraction, and distension profiles of all participants at two-time points of gestation: T1 and T2. The crucial findings were that the HA in square cm at T1 at rest, contraction, and distension was 13.5 (2.1), 12.4(3.7), and 15.1 (2.8), respectively; at T2, 14.5 (2.2), 13.8 (3.4), and 16.5 (3.0), respectively.

**Table 2:** Translabial 3DUS biometry during pregnancy. N = 93

	REST				CONTRACTION				DISTENSION			
	M	SD	Min	Max	M	SD	Min	Max	M	SD	Min	Max
**Antero-posterior T1**	**5.6**	**0.7**	**4.3**	**7.1**	**5.1**	**0.8**	**3.8**	**7.2**	**5.6**	**0.7**	**3.9**	**7.2**
**Antero-posterior T2**	**5.8**	**0.7**	**4.0**	**7.4**	**5.1**	**0.9**	**3.5**	**7.3**	**5.9**	**0.6**	**3.6**	**7.3**
**Antero-posterior T1-T2**	**0.2**	**0.5**	**-1.3**	**1.7**	**0.0**	**0.7**	**-2.7**	**1.2**	**0.2**	**0.6**	**-1.1**	**2.2**
**Transversal T1**	**3.9**	**0.4**	**3.0**	**4.6**	**3.9**	**0.5**	**2.8**	**4.9**	**4.6**	**4.8**	**3.0**	**4.3**
**Transversal T2**	**4.0**	**0.4**	**3.3**	**4.9**	**4.0**	**0.6**	**3.0**	**5.7**	**4.3**	**0.5**	**3.2**	**6.0**
**Transversal T1-T2**	**0.1**	**0.3**	**-0.8**	**0.7**	**0.2**	**0.5**	**-1.1**	**1.5**	**-0.4**	**4.8**	**-3.9**	**1.8**
**Hiatal area T1**	**13.5**	**2.1**	**8.4**	**18.8**	**12.4**	**3.7**	**0.8**	**21.8**	**15.1**	**2.8**	**8.9**	**21.8**
**Hiatal area T2**	**14.5**	**2.2**	**10.3**	**19.2**	**13.8**	**3.4**	**9.1**	**22.8**	**16.5**	**3.0**	**8.9**	**26.4**
**Hiatal area T1-T2**	**1.0**	**1.5**	**-2.7**	**5.1**	**1.3**	**2.4**	**-7.8**	**9.9**	**1.4**	**2.6**	**-4.7**	**11.1**

M: mean; SD: standard deviation; Min: minimum; Max: maximum

T1: 24–28 weeks of gestation; T2: 36–40 weeks of gestation; T1-T2: the interval between T1 to T2

Antero-posterior and Transversal diameter in cm.

Hiatal area in cm square.

Table 3 demonstrates the percentual variation of 3DUS biometry from T1 to T2 pregnancy and during functional maneuvers. The primary feature is the T1-T2 percentual variation of HA from distension to contraction maneuvers, with a mean of -46.8 (SD 28.5).

**Table 3:** Percentual Variation of Translabial 3DUS Biometry during Pregnancy. N = 93

	**% CONTRACTION-REST**				**% DISTENSION-REST**				DISTENSION-CONTRACTION			
	M	SD	Min	Max	M	SD	Min	Max	M	SD	Min	Max
**T1 ANTERO-POSTERIOR**	**-8.4**	**15.8**	**-34.0**	**1.3**	**1.3**	**13.6**	**-29.0**	**42.3**	**12.2**	**16.3**	**-19.3**	**68.2**
**T1 TRANSVERSAL**	**-0.2**	**14.9**	**-34.7**	**19.7**	**19.7**	**12.3**	**-21.0**	**101.0**	**22.3**	**13.0**	**-29.1**	**106.7**
**T1 HIATAL AREA**	**-6.9**	**29.7**	**-93.8**	**12.5**	**12.5**	**20.1**	**-36.6**	**75.5**	**72.6**	**29.3**	**-36.7**	**202.6**
**T2 ANTERO-POSTERIOR**	**-12.4**	**16.0**	**-42.3**	**18.8**	**18.8**	**19.3**	**-38.1**	**50.5**	**18.8**	**19.3**	**-38.1**	**50.5**
**T2 TRANSVERSAL**	**0.0**	**15.4**	**-25.8**	**8.5**	**8.5**	**16.7**	**-31.0**	**62.5**	**8.5**	**16.7**	**-31.0**	**62.5**
**T2 HIATAL AREA**	**-3.2**	**27.5**	**-45.2**	**25.8**	**25.8**	**34.4**	**-46.0**	**152.0**	**25.8**	**34.4**	**-46.0**	**152.0**
**T1-T2 ANTERO-POSTERIOR**	**-4.0**	**12.3**	**-35.4**	**17.5**	**17.5**	**23.5**	**-42.3**	**66.6**	**6.6**	**16.0**	**-27.2**	**54.9**
**T1-T2 TRANSVERSAL**	**0.2**	**13.0**	**-35.6**	**-11.3**	**-11.3**	**12.3**	**-99.5**	**63.9**	**-13.8**	**12.9**	**-105.2**	**47.7**
**T1-T2 HIATAL AREA**	**3.7**	**18.0**	**-40.5**	**13.2**	**13.2**	**37.2**	**-75.5**	**116.5**	**-46.8**	**28.5**	**-195.0**	**92.2**

M: Mean

SD: Standard deviation

Min: minimum value

Max: maximum value

All variables disposed of in %

T1: 24–28 weeks of gestation; T2: 36–40 weeks of gestation

Table 4 demonstrates the Cox univariate and multivariate logistic regression analysis among UI, clinical, and HA measurements.

**Table 4:** Cox univariate and multivariate logistic regression analysis among 6–18 months UI, Clinical, and Hiatal Area measurements. N = 93

	Univariate		Multivariate					
			FULL MODEL			BEST MODEL		
	**RR**	***P***	RR	95%CI	***P***	**Adj.RR**	**95%CI**	***P***
**Gestational Diabetes Mellitus**	**5.44**	**.017**	2.59	0.23–28.91	.440	8.08	1.17–55.8	.034
**Age**	**1.01**	**.852**						
**First trimester Body Mass Index**	**1.07**	**.229**						
**Pregnancy weight gain**	**0.85**	**.046**						
**T1 Gestational age**	**1.15**	**.674**						
**T2 Gestational age**	**0.46**	**.314**						
**Interval T1-T2**	**0.72**	**.322**						
**Pregnancy ICIQ-SF score**	**1.11**	**.073**	1.00	0.58–1.70	.986			
**PS-UI**	**3.60**	**.110**	0.90	0.00-1770	.979			
**Pregnancy ISI score**	**1.40**	**.045**	1.38	0.11–17.78	.803			
**Educational Superior Level**	**0.09**	**.001**	0.17	0.01–3.92	.266			
**Non-Caucasian ethnicity**	**0.03**	**.304**						
**Physical activity in pregnancy**	**0.03**	**.424**						
**Newborn weight**	**1.00**	**.160**	1.00	1.00–1.00	.973			
**Postpartum Follow-up interval**	**1.21**	**.404**						
**Postpartum maternal weight**	**1.01**	**.354**						
**Breastfeeding period**	**0.98**	**.868**						
**Postpartum physical activity**	**0.03**	**.379**						
**Postpartum Diabetes diagnosis**	**0.04**	**.668**						
**T1 at Rest**	**1.03**	***.820***						
**T1 at Contraction**	**1.05**	***.572***						
**T1 at Distension**	**1.06**	***.620***						
**T2 at Rest**	**0.89**	***.482***						
**T2 at Contraction**	**1.12**	***.214***						
**T2 at Distension**	**0.92**	***.529***						
**Variation T1 to T2 at Rest**	**0.74**	***.181***	0.93	0.40–2.16	.868			
**Variation T1 to T2 at Contraction**	**1.12**	***.347***						
**Variation T1 to T2 at Distension**	**0.84**	***.206***						
**% Contraction to Rest T1**	**1.00**	***.591***						
**% Distension to Rest T1**	**1.00**	***.685***						
**% Distension to Contraction T1**	**0.99**	***.732***						
**% Contraction to Rest T2**	**1.01**	***.110***	0.98	0.91–1.05	.561			
**% Distension to Rest T2**	**0.97**	***.090***	0.98	0.91–1.05	.536			
**% Distension to Contraction T2**	**0.97**	***.090***						
**T1-T2% Variation Contract to Rest**	**1.02**	***.111***	1.06	0.97–1.15	.199			
**T1-T2% Var. Distension to Rest**	**0.98**	***.077***	0.96	0.89–1.05	.376	0.96	0.93–0.99	.023
**T1-T2% Var. Distension to Contract**	**1.00**	***.746***						

For univariate Cox Linear Regression, *P*-value:0.20

T1: 24–28 weeks of gestation; T2: 36–40 weeks of gestation

ICIQ-SF: International Consultation on Incontinence Questionnaire - Short Form.

PS-UI: Pregnancy-specific urinary incontinence; Gestational ISI: Incontinence Severity Index during pregnancy

For multivariate logistic regression, *P*-value: 0.05

The best model was obtained after 8 Backward elimination methods.

The percentual Hiatal Area distension-contraction at T2 was excluded for collinearity with other variables.

The maternal weight gain was excluded because the result was not clinically explained.

RR: Relative Risk; Adj RR: Adjusted Relative Risk; 95% CI: 95% Confidence Interval

The significant univariate results, using a *P*-value cutoff of 0.20, were: GDM diagnosis, pregnancy weight gain, transversal diameter at rest, HA T1-T2 at rest, percentual variation of HA from contraction to rest at T2, percentual variation of HA from distension to rest at T2, percentual variation of HA from distension to contraction at T2, percentual variation from contraction to rest of HA from T1-T2, and from distension to rest of HA from T1-T2, PS-UI, pregnancy ICIQ-SF score, pregnancy ISI score, superior education level, and newborn weight. The UI risk was 5.4 times higher in women with positive 75 g-OGTT, decreased with increased weight gain, decreased with an increased variation from T1-T2 in transversal diameter and HA at rest; was 3.6 times higher in women who had PS-UI (*P* < .20). The variables with a *P-value* under 0.20 in the univariate correlation analysis were included in the multivariate linear regression analysis using the backward stepwise method. We did not use the variable weight gain because its clinical interpretation was inconsistent in this study; univariate analysis demonstrates that a higher weight gain was related to a lower RR for 6–18 months UI, a condition not observed clinically. The multivariate logistic regression analysis was performed after adjustments for GDM, PS-UI diagnosis, superior educational level, variation from T1 to T2 of transversal diameter and HA at rest, percentual variation of HA from contraction to rest and from distension to rest at T2; percentual variation of HA from contraction to rest and from distension to rest from T1 to T2; gestational ICIQ-SF and ISI scores; and newborn weight (model 1). In the backward stepwise model, some variables were eliminated, avoiding collinearity, and after adjustments for the newborn weight (model 2, *P*:0.992); for gestational ICIQ-SF (model 3; *P*:0.778); for the percentual of HA variation from contraction to rest at T2 (model 4; *P*:0.576); for PS-UI (model 5; *P*: 0.296); for the variation from T1-T2 of transversal diameter at rest (model 6; *P*: 0.228); for the percentual variation from T1 to T2 of HA from contraction to rest (model 7; *P*:0.093). Adjusted analysis of potential confounders disclosed that UI development was significantly associated with GDM (model 8; Adjusted RR: 8.08; 95%CI (1.17–55.8); *P*:0.034) and the percentual variation from T1 to T2 of the HA from distension to rest (model 8; Adjusted RR:0.96; 95%CI (0.93–0.99); *P*:0.023).

## Discussion

Mild GDM pregnancy, treated with nutritional and healthy lifestyle interventions, is a solid and independent risk factor for 6–18 months of UI development. Second, higher PF Hiatal area distension in the third trimester of pregnancy is a protective factor. Consistent with these findings, our study reports a positive relationship between GDM and increased 6–18 months UI and a negative relationship between higher HA distension and decreased UI. Thus, both markers are opposed, as GDM is a causative agent for UI development. On the other hand, as observed by PF 3DUS, higher HA distensibility is a protective agent.

GDM diagnosis finds a group of women and their offspring at higher risk of diabetes, obesity, and premature cardiovascular disease in the long term [29]. Identifying long-term UI development for mothers with GDM has yet to be fully clarified in the literature. Multivariate regression analyses revealed that GDM, even treated only with healthful dietary patterns and lifestyle intervention, without insulin or oral hypoglycemic drugs therapy, was a solid and independent risk factor for 6–18 months UI (Adjusted RR 8.08; 95% CI 1.17–55.87, *P*:0.034). By different approaches, previous translational studies have demonstrated that GDM models in pregnant rats are enough to cause hyperglycemic myopathy in PFM and RAM [6, 10, 11], structures directly involved in the urinary continence mechanisms. Hence, this association between GDM and future UI risk results from transient hyperglycemia exposure of the muscles involved in the continence mechanisms [4]. GDM is confirmed as a vital risk factor for UI development [4]. Furthermore, our findings add to previous clinical and experimental studies, elaborated, step by step, through translational research, suggesting hyperglycemic myopathy as the underlying mechanism between GDM and UI [4].

In this study, the PF 3DUS biometrical change assessment, from the second to the third trimester of pregnancy, detected an increase in the PF HA distensibility at the end of the third trimester of pregnancy as a protective factor against 6–18 months UI. This unexpected functional observation by 3DUS, identifying a PFM becoming more distensible or complacent at the end of the third trimester of pregnancy, is not previously reported in the literature. The higher PFM distensibility observed by 3DUS, the lower the PF lesion risk and, consequently, the lower the risk of UI development. This higher PFM distensibility at the 3DUS functional image allows us to display its natural protective role against UI development, confirming classical obstetric knowledge. The protective role of higher PFM distensibility pre-delivery is well described and attributed to physiological preparation for vaginal delivery [5–7].

The strengths of our study include its longitudinal design with a 1–2-year follow-up rate, considering GDM as a transient condition with lifelong sequels. All PFM 3DUS was done by the same author, with blinded offline analyses of clinical data and US raw data. By enrolling women during pregnancy, we provided selection criteria such as previous parity, labor induction and second period of labor, and vaginal delivery, which are considered relevant postpartum UI risk factors. In fact, the 93 analyzed women underwent a planned, elective C-section. All GDM patients treated with hypoglycemic drugs were excluded to register a possible maternal PFM physiological adaptation to vaginal delivery. This analysis allows us to obtain the best model after eight backward models of multivariate analyses confirming that GDM, even treated with nutritional and lifestyle intervention, is a solid predictor of 6–18 months UI despite the short period of hyperglycemic status exposure during pregnancy. In this multivariate analysis, the higher PFM distensibility at the end of pregnancy enables us to register a possible maternal PFM physiological adaptation to vaginal delivery.

Limitations: First, our results may not be generalizable and do not apply to other populations, including multiparous or GDM women treated with insulin or hyperglycemic drugs. Second, our strict inclusion criteria of GDM women-only treatment with nutritional and lifestyle interventions probably removed essential risk factors for long-term UI. Third, we do not collect data on the prepregnancy and first trimester due to the first enrollment in the second trimester of gestation.

Our results collectively suggested that universal GDM diagnosis and regular PFM 3DUS functional analysis are good clinical indexes for later UI prediction (concerning GDM diagnosis) or UI protective prediction (concerning higher distensibility of HA). In addition, pregnancy serves as a screening tool for future health risks, opening a window of opportunities to prevent two relevant diseases of our time: GDM and UI [28]. Predicting postpartum UI during pregnancy, with clinical markers like GDM and Pelvic floor biometrical changes by 3DUS, are opportunity to propose clinical interventions to mitigate or prevent postnatal urinary disorders such as UI.

## Conclusion

Our results provide objective data that GDM-pregnancies are positively related to increasing the risk of 6–18 months of UI development. Thus, GDM exposure, only with nutritional and healthy lifestyle counseling, is a solid and independent risk predictor for 6–18 months UI, as observed in this cohort of nulliparous women submitted to a planned C-section. While further studies are required to elucidate the mechanism, mild GDM may represent a modifiable risk factor for 6–18 months UI. Opposite results were found for higher Hiatal area distension to rest from the second to the third trimester, related to decreasing the risk of 6–18 months UI. Further studies may determine the best pelvic floor intervention approach during GDM pregnancy to prevent later UI.

## Data Availability

Due to an ongoing study with preliminary results, the datasets used and/or analyzed during the current study are available from the corresponding author upon reasonable request.
